# *APC* and *MUTYH* Analysis in FAP Patients: A Novel Mutation in *APC* Gene and Genotype-Phenotype Correlation

**DOI:** 10.3390/genes9070322

**Published:** 2018-06-27

**Authors:** Giovanna D’Elia, Gemma Caliendo, Amelia Casamassimi, Michele Cioffi, Anna Maria Molinari, Maria Teresa Vietri

**Affiliations:** 1 Department of Precision Medicine, University of Campania “Luigi Vanvitelli”, 80138 Naples, Italy; giovannadelia@alice.it (G.D.); gemma.caliendo@unicampania.it (G.C.); annamaria.molinari@unicampania.it (A.M.M.); mariateresa.vietri@unicampania.it (M.T.V.); 2 Department of Medical, Surgical, Neurological, Aging and Metabolic Sciences, University of Campania “Luigi Vanvitelli”, 80138 Naples, Italy; michele.cioffi@unicampania.it

**Keywords:** familial adenomatous polyposis, *APC* gene, *MUTYH* gene, mutational analysis, genotype-phenotype correlation, familial cancer, extracolonic manifestation

## Abstract

*APC* and *MUTYH* genes are mutated in 70–90% and 10–30% of familial adenomatous polyposis cases (FAP) respectively. An association between mutation localization and FAP clinical phenotype is reported. The aims of this study were to determine *APC* and *MUTYH* mutational status in a small cohort of FAP patients and to evaluate the genotype-phenotype correlation in mutated patients. Here, we report the identification of a novel *APC* germline mutation, c.510_511insA. Overall, mutational analysis showed pathogenic mutations in 6/10 patients: 5/10 in *APC* and 1/10 in *MUTYH*. Additionally, we found three variants of unknown significance in *MUTYH* gene that showed no evidence of possible splicing defects by in silico analysis. Molecular analysis was also extended to family members of mutated patients. A genotype-phenotype correlation was observed for colonic signs whereas a variation of disease onset age was revealed for the same mutation. Moreover, we found an intrafamilial variability of FAP onset age. Regarding extracolonic manifestations, the development of desmoid tumors was related to surgery and not to mutation position, while a genotype-phenotype correspondence was observed for the onset of thyroid or gastric cancer. These findings can be useful in association to clinical data for early surveillance and suitable treatment of FAP patients.

## 1. Introduction

Familial adenomatous polyposis (FAP) is a hereditary disorder characterized by a large number of precancerous polyps initiating to grow in childhood and adolescence. These polyps transform into colorectal carcinoma during the fourth and fifth decades of life [1]. Several clinical variants of the FAP phenotype have been described, based on the number of colorectal polyps and onset age [2]. Thus, FAP phenotype was primarily categorized into profuse or aggressive FAP, attenuated FAP and intermediate FAP [3]. In particular, profuse or aggressive FAP is characterized by the presence of hundreds to thousands of adenomatous polyps throughout the colon and the rectum; moreover, about half patients develop adenomas by the age of 15 years. Colorectal cancer (CRC) inevitably occurs at an earlier age than sporadic CRC. Intermediate FAP is characterized by the presence of hundreds of polyps that develop around the second-third decade of life. CRC occurs at approximately 40 years. Attenuated FAP displays a mild disease course, characterized by a reduced number of polyps (10–100), frequently colon right-sided distributed, and lower CRC risk, which manifests itself at an advanced age compared to the profuse FAP [4]. Moreover, different clinical phenotypes associated to extracolonic manifestations were observed in FAP patients [5].

Monoallelic mutations in the adenomatous polyposis coli (*APC*) gene were firstly indicated as causing FAP with an autosomal dominant pattern of inheritance [4]. Moreover, biallelic mutations in mutY homolog (*MUTYH*) gene were found to be associated, with an autosomal recessive pattern of inheritance, in a colorectal polyposis referred as MAP (*MUTYH*-associated polyposis). The disease phenotype of MAP is relatively mild, like the attenuated form of FAP, showing fewer than 100 adenomas and an average age at diagnosis of about 45 years. In some patients with MAP the occurrence of serrated polyps and development of extracolonic manifestations can be found [6].

Overall, germline mutations in *APC* gene have been found to be responsible for 70–90% of FAP cases [7]. *MUTYH* mutations have been reported in 10–30% of FAP patients that are negative for *APC* mutation [8].

To date, at least 3000 different germline pathogenic *APC* mutations [9] and 1000 *MUTYH* mutations [10] in FAP patients have been described. The majority of *APC* mutations are nonsense mutations or small insertions or deletions that lead to a truncated protein. Otherwise, *MUTYH* pathogenic mutations are mostly missense substitutions followed by a minority of splice site or truncating mutations. Furthermore, a low prevalence of rearrangements has also been reported in *APC* and *MUTYH* genes [11,12].

In the *APC* gene there are two mutational hotspots, localized at codons 1061 and 1309, at the beginning of exon 15 [4].

The genotypic understanding of the *APC* mutation is likely to be clinically relevant to the phenotypic presentation. Indeed, several studies reported an association between the localization of *APC* mutation and the phenotype in FAP patients [13]. Age of onset, number of polyps and occurrence of extracolonic manifestations appear to correlate with specific mutation sites [14]. However, some studies reported a wide variation of genotype-phenotype correlations in patients with the same mutation [15].

Genotype-phenotype associations have been described also for *MUTYH* mutations. Several studies have shown that patients with biallelic Y179C mutations display a more aggressive phenotype compared to patients with biallelic G396D mutations, in accordance with the functionality assays reporting a greater reduction of *MUTYH* glycosylase activity for p.Y179C than p.G396D alleles [16].

Today, more than 400 and 300 variants of unknown significance (UVs) have been identified in *APC* and *MUTYH* genes respectively [9,10]. Classification of UVs as either deleterious or benign is an important process for appropriate medical management. Currently, many in silico algorithms can be used to assess the effects of UVs on mRNA splicing or protein function. Additionally, in vivo and in vitro studies and co-segregation analyses may support the clinical importance of UVs [17].

The aims of this study were: (1) to determine *APC* and *MUTYH* mutational status in a small cohort of FAP patients and to extend genetic analysis to their families; (2) to evaluate the genotype-phenotype correlation in mutated patients; (3) to assess the pathogenetic significance of the identified variants. 

Here, we describe the identification and characterization of a novel germline mutation in the *APC* gene in a patient affected with FAP and in her family.

## 2. Materials and Methods

### 2.1. Patients

This study was performed in accordance with guidelines of the local Ethics Committee (AOU—University of Campania—Luigi Vanvitelli) and with the World Medical Association Helsinki Declaration, adopted in 1964, and amended in 1975, 1983, 1989, 1996 and 2000 (WMA 1998) (DH e B1/2). Genomic DNA samples were retrieved from patients, family members and healthy subjects who had been referred for genetic testing at our laboratory. Informed consent was obtained from all participants. In this study, we enrolled 10 Italian patients with FAP (females (F) = 7; males (M) = 3), with an age interval of 22–56 years. The median age at polyp diagnosis was 36 years. The main clinical and histopathological data were acquired by genetic counselling. Mutational analysis was extended to family members of mutated patients. As control, 15 healthy subjects, (age 25–75 years) from the same geographical area were selected. They were all cancer-free at the time of blood donation.

### 2.2. Mutation Analysis

Peripheral blood samples were collected from all patients. Genomic DNA was extracted using the Wizard Genomic DNA purification kit (Promega, Fitchburg, WI, USA) according to the manufacturer’s instructions.

Mutational analysis was performed amplifying 15 exons and adjacent intronic regions of *APC* gene, as previously reported [18]. All 16 exons of the *MUTYH* gene and the flanking splice site regions were amplified using a set of ten primer pairs [19].

All PCR products were sequenced on both strands using the ABI Prism di-Deoxy Terminator Cycle Sequencing kit in the ABI 9700 thermal cycler and an ABI Prism 3100 automatic sequencer (both from Life Technologies, Carlsbad, CA, USA). The results were analyzed using Mutation Surveyor^®^ software, version 3.24 (Softgenetics, State College, PA, USA).

The novel mutation was named and referred in the text according to the nomenclature used by Human Genome Variation Society [20] and to the descriptions suggested by den Dunnen and Antonarakis [21].

### 2.3. In Silico Analysis

To evaluate a potential alternative splicing effect of the variants found, the integrated software Alamut V.2.11 (January 2018) (Interactive Biosoftware, Rouen, France. Available at http://www.interactive-biosoftware.com). This software included four prediction algorithms viz. SpliceSiteFinder-like, MaxEntScan, NNSPLICE and GeneSplicer. The genomic sequence spanning the individual mutations and nearby exons was submitted according to the guidelines of each program and default settings were used in all predictions. A variation of more than 10% in at least two algorithms was considered as having an effect on splicing [22]. 

## 3. Results

Mutational analysis showed pathogenic mutations in 6/10 (60%) patients. In this small cohort, five patients (50%) displayed a mutation in APC whereas one patient (10%) had a biallelic mutation in *MUTYH*. Out of five mutated patients in APC, two had the same mutation.

Otherwise, one of the APC mutations was a novel mutation, specifically c.510_511insA (p.Ser171LysfsX6) mutation, another one had been previously identified in our laboratory, the c.1605_1606delTG (p.Ser535SerfsX3) mutation, while two of them were already described mutations, c.1192_1193delAA (p.Lys398GlufsX5) and c.3927_3931delAAAGA (p.Glu1309AspfsX4).

**Figure 1:**
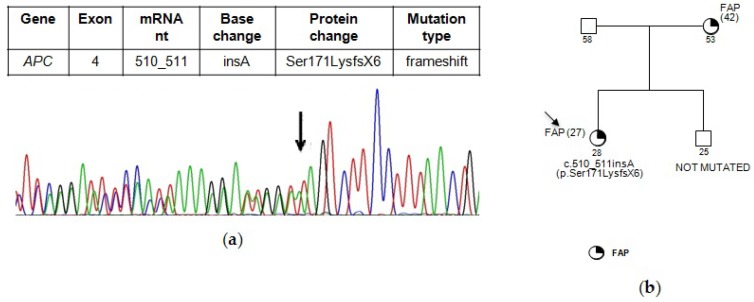
(**a**) Partial electropherogram of *APC* exon 4. The novel mutation identified c.510_511insA (p.Ser171LysfsX6) results in the introduction of a stop codon at amino acid position 176. (**b**) Pedigree of family carrying the *APC* mutation c.510_511insA (p.Ser171LysfsX6). In brackets the age at diagnosis is indicated.

The novel mutation c.510_511insA (p.Ser171LysfsX6) has not been previously reported in published literature or in any database. This frameshift mutation, localized in exon 4, consists of an adenine insertion between nucleotide 510 and 511, which results in the introduction of a stop codon at amino acid position 176 (Figure 1a). It was identified in a 28-year-old woman affected with intermediate FAP diagnosed at the age of 27. The genotype analysis was performed also in the unaffected brother of 25 years, reporting a wild type profiling of APC (Figure 1b).

**Figure 2:**
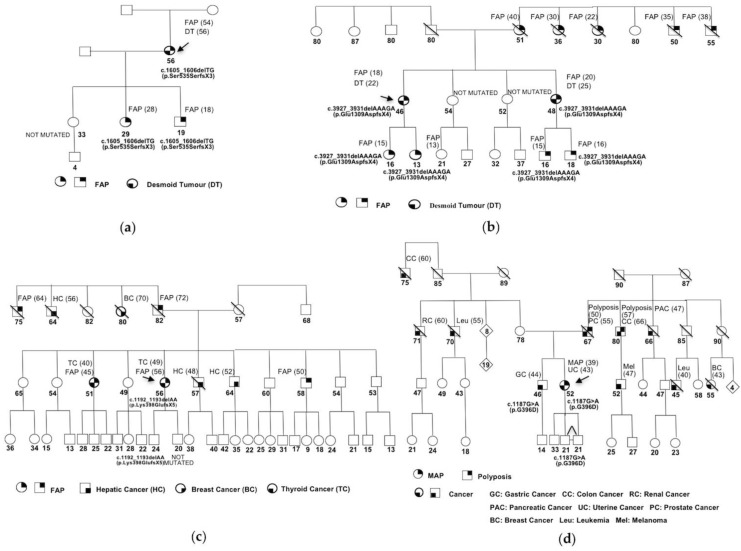
Pedigrees of familial adenomatous polyposis cases (FAP) and *MUTYH*-associated polyposis (MAP) families with extracolonic manifestations. The ages at diagnosis are indicated in brackets. (**a**) Family carrying the *APC* mutation c.1605_1606delTG (p.Ser535SerfsX3); (**b**) Family carrying the *APC* mutation c.3927_3931delAAAGA (p.Glu1309AspfsX4); (**c**) Family carrying the *APC* mutation c.1192_1193delAA (p.Lys398GlufsX5); (**d**) Family carrying the *MUTYH* mutation c.1187G>A (p.G396D).

The previously described mutation c.1605_1606delTG (p.Ser535SerfsX3) occurs in exon 12. It was identified in a woman affected with intermediate FAP, diagnosed at age 54, and in two of her children with FAP, diagnosed at age of 18 and 28 respectively. The healthy daughter did not have this mutation. The proband had surgical treatment with restorative proctocolectomy and ileal J-pouch at the age of 54 years. Two years after the pouch procedure the proband developed desmoid tumors (Figure 2a).

The mutation c.3927_3931delAAAGA (p.Glu1309AspfsX4) is localized in exon 15 and was observed in two patients. The first is a 35 years-old patient with profuse FAP. Unfortunately, none family member was available for genetic testing. Thus, the lack of useful DNA samples has implicated that transmission of this mutation is uncertain in the patient’s family.

The second patient carried of the c.3927_3931delAAAGA (p.Glu1309AspfsX4) mutation is a 46 years-old woman. The patient, affected with profuse FAP, had surgical treatment with colectomy at the age of 18 years. Four years after the pouch procedure the proband developed desmoid tumors. She reported other FAP cases in the family (Figure 2b). The analysis was performed in the daughters of 13 and 16 years affected with FAP, which showed the mutation. Furthermore, mutational analysis was conducted in the sister of 48 years affected whit FAP diagnosed at 20 years, and in her children, of 16 and 18 years, with FAP. The sister and her children showed the mutation. The sister developed desmoid tumors five years after the colectomy. In addition, the analysis was extended to the unaffected sisters of 54 and 52 years that reported wild type profiling of APC.

The mutation c.1192_1193delAA (p.Lys398GlufsX5), localized in exon 9, was found in a 56-years-old woman who was diagnosed with attenuated FAP and thyroid cancer. The pedigree, reported in Figure 2c, shows other cases of FAP in the family. Particularly, her sister, 51 years-old, was affected with FAP and thyroid cancer, diagnosed at age of 45 and 40 years respectively, and her brother, 58 years-old, was affected with FAP, diagnosed at age of 50 years. Molecular analysis was performed only in the unaffected sons of 20 and 24 years and revealed that the APC mutation was carried only by the son of 24 years, while the son of 20 years reported a wild type profiling.

The homozygous *MUTYH* mutation c.1187G>A (p.G396D) was found in a 52-years-old woman affected with MAP, diagnosed at age 39. The analysis was performed in the brother of 46 years affected with gastric cancer that reported the same homozygous mutation. Furthermore, mutational analysis was conducted in her children, a male of 33 years and two twins of 21 years. The twins showed the heterozygous mutation, whereas her son had a wild type profile. The pedigree showing other cancer cases in the family is reported in Figure 2d.

Moreover, in *MUTYH* gene, 3 patients reported the same UV c.1014G>C (p.Q324H). One of the patients, in addition to this homozygous variant, showed two heterozygous UVs, c.157+30A>G (IVS2+30 A>G) and c.813C>T (p.D271D).

The already described UVs, c.157+30A>G (IVS2+30 A>G) and c.813C>T (p.D271D), have not yet been previously tested with prediction programs. To evaluate potential splicing effects, we conducted the in silico analysis using four different splice site prediction programs which predict changes in splice site strength. The threshold employed was a variation between the wild type and the mutation score of more than 10% in at least two different algorithms [22]. No splicing alterations were predicted for the variants c.157+30A>G (IVS2+30 A>G) and c.813C>T (p.D271D) (see Table 1).

Table S1 summarizes all the clinical data, FAP phenotype and *APC*/*MUTYH* mutational status of probands. Figure S1 schematizes the correlation between genotype and FAP phenotype and between localization of the *APC* mutations and extracolonic manifestations, as reported in literature. Moreover, the localization of *APC* mutations found in our patients is shown. 

## 4. Discussion

Despite the small group of patients analyzed for mutations in *APC* and *MUTYH* genes, we report the identification of a novel germline mutation in *APC* gene, c.510_511insA (p.Ser171LysfsX6). Noteworthy, this frameshift mutation is localized in exon 4 and it introduces a stop codon at amino acid position 176 of the *APC* gene product. Since most of the normal protein is lost in the resulting polypeptide, it is conceivable that this mutation is deleterious. Overall, our mutational analysis has detected pathogenic mutations in 6/10 (60%) patients. Specifically, five patients (50%) displayed *APC* mutations whereas one patient (10%) had a *MUTYH* biallelic mutation. Thus, our detection rate was slightly lower than literature data that report a percentage of 70–90% for *APC* and 10–30% for *MUTYH* [8].

The *APC* “mutation cluster region” encompassing codons 1250–1464 is a region with a high mutation rate, with mutational hotspots at codons 1309 and 1061, accounting for approximately 17% and 11% of all germline *APC* mutations, respectively [4]. Interestingly, the c.3927_3931delAAAGA (p.Glu1309AspfsX4) mutation, identified in 2/5 probands, resides in a mutational hotspot, at codon 1309.

A correlation between the *APC* mutation localization and the clinical manifestations was observed in many studies [2]. Regarding the colonic manifestations, profuse or aggressive FAP was related to mutations at codons 1250–1464, mainly at codon 1309. Patients carrying mutations at codon 1309 in particular tend to develop adenomas more frequently and at an earlier age than patients with other mutations [24]. Otherwise, intermediate FAP was associated with mutations localized in the codon interval 157–1595 but excluding codon 1309. Finally, attenuated FAP was associated with mutations outside the 157–1595 interval and in the alternatively spliced region of exon 9 (new codons 213–412) [25]. Of note, our findings are in agreement with the known genotype-phenotype correlation.

However, concerning the age of intestinal symptom onset, the same mutation at codon 1309 has been associated with a large variation, which may be caused by modifier genes or other endogenous or exogenous factors [15]. Accordingly, we also observed a consistent age variation in the proband carriers of the mutation c.3927_3931delAAAGA (p.Glu1309AspfsX4). Indeed, the first proband and her family displayed an early age of onset of FAP, while the second proband developed FAP at a higher age (35 years-old) (Figure 2b). Unfortunately, since we do not have data concerning the family, we cannot advance additional considerations.

Besides, we observed an intra-familial variability of FAP onset also in the other three families analyzed (Figure 1b and Figure 2a–c)

FAP patients often display extracolonic manifestations, including desmoid tumors, osteomas, dental abnormalities, congenital hypertrophy of the retinal pigment epithelium, lipomas, epidermoid cysts and upper gastrointestinal polyps and/or extracolonic malignancies [2]. A relationship between frequencies of extracolonic manifestations and the positions of the *APC* germline mutation in patients with FAP is also known [26]. Interestingly, in our study 3/5 *APC* mutated probands and their families exhibited extracolonic manifestations, two with desmoid tumors and one with thyroid cancer. Specifically, patients with the mutations c.1605_1606delTG (p.Ser535SerfsX3) and c.3927_3931delAAAGA (p.Glu1309AspfsX4) were affected with FAP and desmoid tumors, developed after restorative proctocolectomy, consistent with previous reports [27] (Figure 2a–b). Desmoid tumors were often associated with mutations between *APC* codons 1445 and 2011 [2]. However, our study has not confirmed this association since these detected mutations are localized elsewhere (Figure S1). In concordance with our results, previous studies with large cohorts reported the failure of this association, suggesting that also mutations localized in other positions could be associated with desmoid tumor development [2]. Otherwise, some studies have suggested the likelihood that specific modifier genes exist, independent of *APC*, whose variation increases the risk of desmoid tumors in FAP patients. Such genes are likely to provide an even stronger stimulus to development of this phenotype than the localization of the *APC* mutation [28,29]. Furthermore, other risk factors, such as pregnancy, surgical trauma and positive family history for desmoid tumors could also be related with this extracolonic sign [30]. Consistently, our patients developed desmoid tumors not later than five years after colectomy; since the median interval between the surgery and the diagnosis of desmoid is from two to five years [27], all patients undergoing surgery are potentially at risk of developing desmoid tumor in that time period.

Additionally, the patient with c.1192_1193delAA (p.Lys398GlufsX5) mutation and her sister are also affected with thyroid cancer. Thyroid carcinoma is the third most common malignancy associated with FAP, with a low developing lifetime risk (2–3%). Thyroid tumors have been related to mutations between codons 140 and 1309 [27]; accordingly, the c.1192_1193delAA (p.Lys398GlufsX5) mutation is localized at the codon 398 (Figure 2c).

In *MUTYH* gene, we detected the mutation c.1187G>A (p.G396D) in a woman affected with MAP. This mutation is the most common in polyposis patients [31]. It is localized in a highly conserved amino acid region in *MUTYH* and has been reported to cause a reduction in the capacity of binding its substrate and impairment of glycosylase activity [32]. This homozygous mutation was detected in the proband with MAP, in her brother with gastric cancer and in heterozygosis in her twin children (Figure 2d). Gastric cancer is one of the extracolonic manifestations of MAP, found in patients with *MUTYH* biallelic mutations [33]. Thus, our result also support a correlation between the mutation c.1187G>A (p.G396D) and gastric cancer.

The presence of polyposis in the patient’s father and uncle could be due to consanguinity but we are not aware of it. Furthermore, the occurrence of other tumors in the family suggests the possible existence of mutations in other genes.

Furthermore, we detected the *MUTYH* UV c.1014G>C (p.Q324H) in 3/3 patients with attenuated FAP, 1/3 homozygous for this variant. Remarkably, a case-control study was performed for p.Q324H variant and any difference between cases and controls was found. Furthermore, this variant was reported as not potentially affecting protein function by the in silico analysis [23]. However, in vitro studies showed that p.Q324H is 36% less active than wild type allele [34]. Because of these conflicting data, it is still reported as UV.

Another patient showed two UVs, c.157+30A>G (IVS2+30 A>G) and c.813C>T (p.D271D). In silico analyses indicate that these variants do not affect splicing (Table 1); however, the evaluation of their pathogenetic significance needs to be corroborated by further experimental evidences.

Currently, mutational analysis of *APC* and *MUTYH* genes is relevant in FAP patients for the identification of disease causing mutations, allowing the extension of genetic testing to family members of mutated patients. In order to carry out an early surveillance and a suitable treatment of FAP patients, a detailed genetic testing with a genotype-phenotype correlation would be very useful in association to clinical data. Certainly, *APC* and *MUTYH* are not the only genes predisposing to the familial polyposis. Indeed, recent literature indicates that also other genes may be involved, like *NTHL1*, *POLD1* and *POLE* genes, even though they are able to explain only a small proportion of cases [35,36]. Moreover, new candidate genes are continually discovered by exome sequencing and are currently subject to further investigation before their use in the diagnosis of these patients [37]. Thus, mutational analysis of these newly identified genes will be the next step in *APC* and *MUTYH* negative samples of our small group of patients.

Regarding the age for the onset of clinical surveillance, the established guidelines suggest that classical FAP patients should start endoscopic surveillance from the early teens, while attenuated FAP and MAP families at the age of 18–20. In FAP cases sigmoidoscopy screening should be carried out every two years; once adenomas are detected, total colonoscopy should be carried out annually until colectomy is planned. In attenuated FAP and MAP cases colonoscopy is recommended instead of sigmoidoscopy, with screening every two years; once adenomas are detected, colonoscopy should be carried out annually [38,39]. Even after a total colectomy, an endoscopic/colonoscopic procedure is recommended every six to 12 months after surgery to assess the anastomosis site, pouch, and residual rectum [5].

## Figures and Tables

**Table 1:** In silico study of *MUTYH* variants. The thresholds represent the score predicted for wild type sequence/score predicted for the mutated sequence. Scores indicate the values for splice donor (SD) or splice acceptor (SA) sites, respectively. Changes relative to wild type sequences are indicated in %.

Designation	Exon/Intron	PolyPhen	SIFT	Splice Site Finder (0–100)	MaxEntScan (0–12)	NNSPLICE (0–1)	GeneSplicer (0–24)	Ref.
IVS2+30 A>G (c.157+30 A>G)	2	-	-	SD:86,16/86,16	SD:9,33/9,33	SD:0,99/0,99	SD:4,35/4,40 (+1.3%)	-
D271D (c.813 C>T)	10	-	-	SA:86,80/86,80	SA:9,49/9,49	SA:0,97/0,97	SA:6,43/6,29 (−2.2%)	-
Q324H (c.1014G>C)	12	Benign (score 1.409)	Tolerated	-	-	-	-	[23]

## Supplementary Materials

The following are available online at http://www.mdpi.com/2073-4425/9/7/322/s1. Table S1: Clinical data, mutations and UVs of *APC* and *MUTYH* in FAP probands. Figure S1: Localization of *APC* mutations, genotype-phenotype correlation and occurrence of extra-colonic manifestation.
