# Supplementary material for: APC and MUTYH Analysis in FAP Patients: A Novel Mutation in APC Gene and Genotype-Phenotype Correlation
Source: Genes (Basel). 2018 Jun 27;9(7):322. doi: 10.3390/genes9070322 (PMC6071208; doi:10.3390/genes9070322)
Supplement: Supplementary file 1 [file genes-09-00322-s001.zip › Figure S1.doc]

**Figure S1.** Localization of APC mutations, genotype-phenotype correlation and occurrence of extra-colonic manifestation.
