# Supplementary material for: APC and MUTYH Analysis in FAP Patients: A Novel Mutation in APC Gene and Genotype-Phenotype Correlation
Source: Genes (Basel). 2018 Jun 27;9(7):322. doi: 10.3390/genes9070322 (PMC6071208; doi:10.3390/genes9070322)
Supplement: Supplementary file 1 [file genes-09-00322-s001.zip › Table S1.doc]

**Table S1.** Clinical data, mutations and UVs of APC and MUTYH in FAP probands.

| **PROBAND** | **AGE** | **AGE AT DIAGNOSIS** | **FAP PHENOTYPE** | **NUMBER OF POLYPS** | **EXTRACOLONIC MANIFESTATIONS** | **FAMILY HISTORY** | **APC MUTATIONS** | **MUTYH**  **MUTATIONS** | **MUTYH UVs** |
| --- | --- | --- | --- | --- | --- | --- | --- | --- | --- |
| 1 | 28 | 27 | Intermediate FAP | > 100 | - | Yes | c.510_511insA (p.Ser171LysfsX6) | - | - |
| 2 | 56 | 56 | Attenuated FAP | < 100 | Thyroid cancer | Yes | c.1192_1193delAA (p.Lys398GlufsX5) | - | - |
| 3 | 56 | 54 | Intermediate FAP | > 100 | Desmoid tumor | Yes | c.1605_1606delTG (p.Ser535SerfsX3) | - | - |
| 4 | 35 | 35 | Profuse FAP | > 1000 | - | Yes | c.3927_3931delAAAGA (p.Glu1309AspfsX4) | - | - |
| 5 | 46 | 18 | Profuse FAP | > 1000 | Desmoid tumor | Yes | c.3927_3931delAAAGA (p.Glu1309AspfsX4) | - | - |
| 6 | 52 | 39 | MAP | < 100 | - | Yes | - | G396D (c.1187G>A)  homozygous | - |
| 7 | 41 | 40 | Attenuated FAP | < 100 | - | Yes | - | - | Q324H (c.1014G>C)  heterozygous |
| 8 | 24 | 18 | Attenuated FAP | < 100 | - | Yes | - | - | Q324H (c.1014G>C)  heterozygous |
| 9 | 54 | 54 | Attenuated FAP | < 100 | - | Yes | - | - | Q324H (c.1014G>C)  homozygous  IVS2+30 A>G (c.157+30 A>G)  D271D  (c.813 C>T) |
| 10 | 22 | 22 | Attenuated FAP | < 100 | - | Yes | - | - | - |
